# Dirty Mass sign of abdominal imaging: A case of silent pneumoperitoneum

**DOI:** 10.1016/j.radcr.2024.02.012

**Published:** 2024-02-22

**Authors:** Garrett Coleman, Hasan Khan, Anthony D'Angelo, Zahra Aktar, Irfan Masood

**Affiliations:** a John Sealy School of Medicine, The University of Texas Medical Branch, Galveston, TX, USA; b Department of Radiology, The University of Texas Medical Branch, Galveston, TX, USA

**Keywords:** Dirty Mass, Dirty Mass sign, Pneumoretroperitoneum, Colonic perforation, Retroperitoneum

## Abstract

Pneumoperitoneum is a common complication after penetrating abdominal trauma, gastric ulcer, or colitis in which free air is present in the peritoneal or retroperitoneal space. Sole pneumoretroperitoneum, which refers to gas in the retroperitoneal space, is a rare entity, and when significant, results in a characteristic radiographic sign known as “Dirty Mass.” Common causes include penetrating trauma or perforation of the retroperitoneal portions of the gastrointestinal tract (duodenum, ascending colon, descending colon, and rectum). Our case describes a 59-year-old female admitted for sudden onset RLQ abdominal pain with Dirty Mass sign on abdominal KUB. Early recognition of these key radiographic findings accelerates management and reduces the risk of developing complications.

## Introduction

Gastrointestinal perforation can result in the spillage of bowel contents into the surrounding peritoneal and retroperitoneal spaces, which often leads to secondary bacterial peritonitis. A common imaging finding is pneumoperitoneum, or presence of air in the peritoneal cavity on radiograph. Less commonly, bowel perforation may also occur into the retroperitoneal space, resulting in pneumoretroperitoneum and a dirty mass sign on radiograph [1]. Diagnosis of gastrointestinal perforation can be obtained with CT scan, which is most sensitive and specific for diagnosing colonic perforation, as well as visualizing the bowel contents in the extraluminal space [2].

## Case presentation

We present the case of a 59-year-old female with past medical history of atrial fibrillation, hypertension, hyperlipidemia, hypothyroidism, and perforated diverticulitis status post diverting colectomy with Hartmann pouch which was reversed 6 years ago. She presented to the emergency department with insidious right lower quadrant abdominal pain that worsened in the past 2 hours, nausea, and chills. She denied vomiting, changes in bowel movements, or fevers. She has had chronic abdominal pain since her colostomy reversal, but this time it was significantly worse in intensity. She was previously seen in the emergency department 1 month ago for left upper quadrant pain secondary to colonic stricture and colitis.

**Fig. 1:**
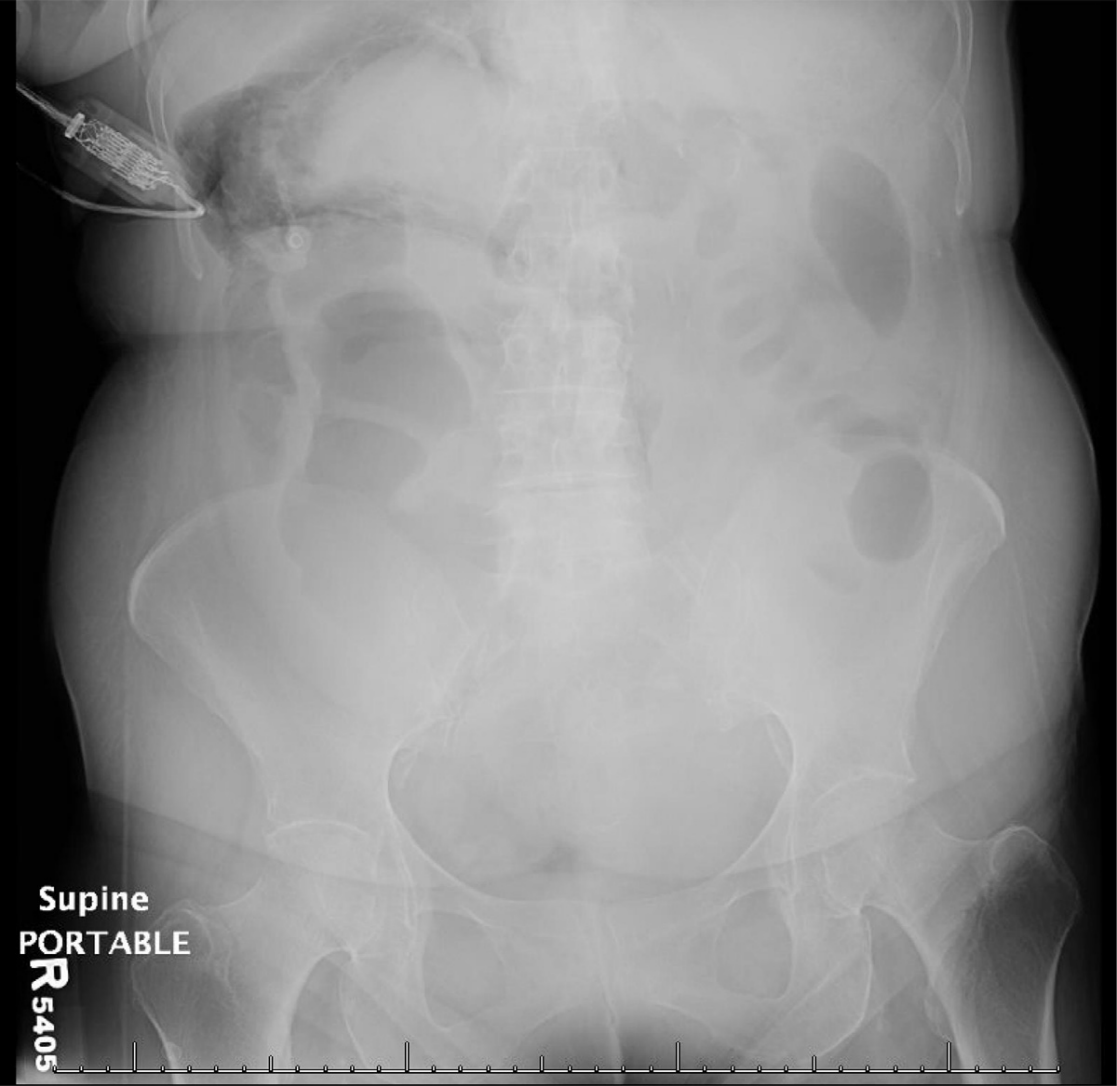
Supine AP view of the abdomen and pelvis with atypical collection of air in the right upper quadrant compatible with “Dirty Mass sign,” outlining the superior pole of the right kidney, findings suggestive of isolated retroperitoneal air.

**Fig. 2:**
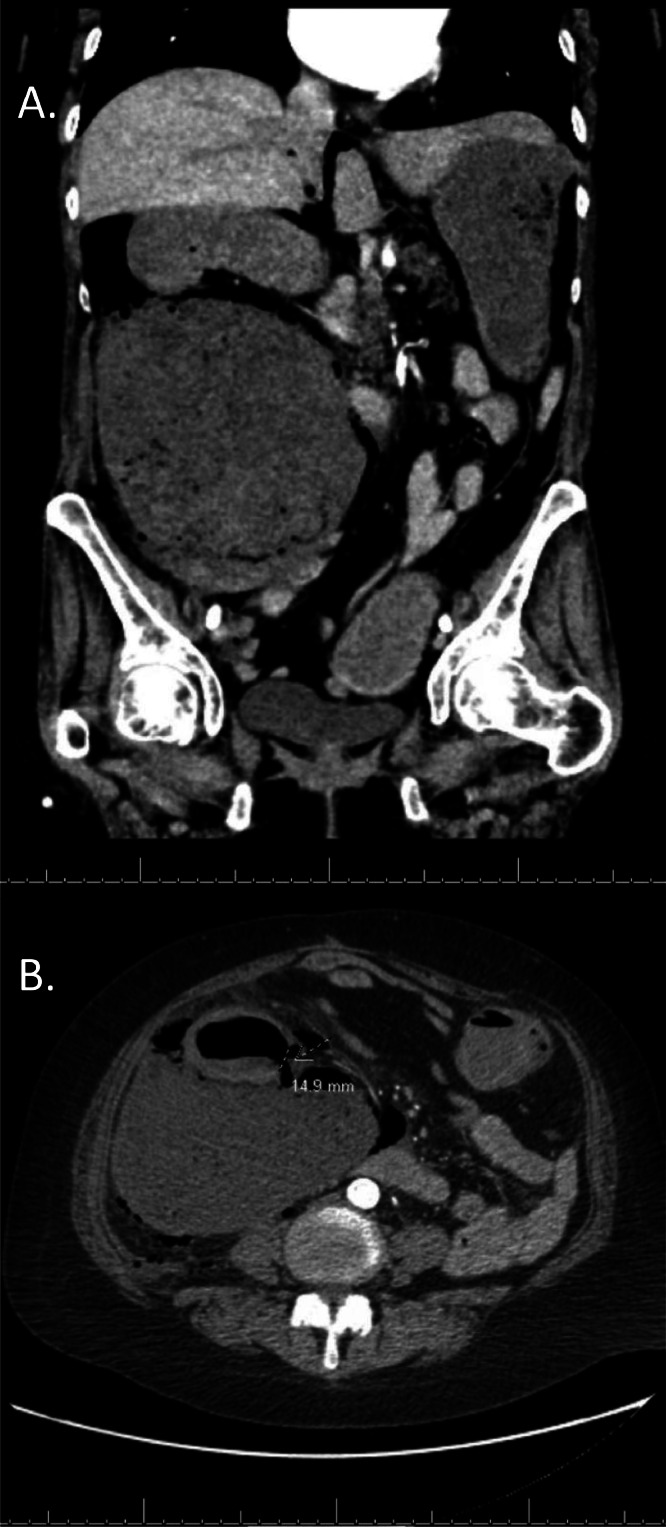
(A) Coronal CT image shows atypical collection of air in the right upper quadrant and (B) axial CT image reveals perforation at the posterior medial wall of the proximal ascending colon.

On physical exam, there was tenderness to palpation in the right and left lower quadrants without rebound tenderness. An abdominal X-ray was first obtained, which revealed “Dirty Mass” sign, from an atypical collection of air in the right upper quadrant, outlining the superior pole of the right kidney (Fig. 1). CT abdomen pelvis was subsequently performed with IV contrast, which showed a 1.5 cm perforation at the posterior medial wall of the proximal ascending colon with leakage of large volume colonic content and enteric gas into the retroperitoneal space (Fig. 2). The patient was referred to surgery and started NPO with IV fluids. Ultimately, she required multiple exploratory laparotomies with right hemicolectomy for cecal/ascending colon perforation, as well as multiple takebacks with Vicryl MESH and skin-only closure. The patient was discharged to a long-term acute care facility.

## Discussion

Gastrointestinal perforation composes a significant percentage of all acute abdomen cases, which itself contributes to 40% of all emergency-surgical hospital admissions [3]. It is a common cause of secondary bacterial peritonitis which has mortality rates ranging from 30% to 50% [4]. This high mortality rate demonstrates the importance of identifying bowel perforations rapidly so that treatment can be initiated.

Pneumoretroperitoneum is the presence of gas within the retroperitoneal space. The cause of pneumoretroperitoneum is always pathological and can occur secondary to residual air following retroperitoneal surgery or from a perforated retroperitoneal hollow viscus in the duodenum (peptic ulcer disease, blunt/penetrating abdominal trauma, endoscopy, endoscopic retrograde cholangiopancreatography), the ascending/descending colon (endoscopy, colorectal carcinoma, diverticulitis, and ischemic colitis), or the rectum (endoscopy, trauma, foreign body insertion, transanal excision of rectal carcinoma) [5], [6], [7], [8], [9]. Patients with pneumoretroperitoneum present with “acute abdomen” and its subsequent abdominal pain, nausea, vomiting, decreased bowel function, and/or fever. On physical exam, tenderness to palpation is present but difficult to localize. The patient's condition may deteriorate to peritonitis and/or sepsis [10].

Pneumoretroperitoneum can be detected on transabdominal ultrasound [11] or abdominal radiograph [12] but is ultimately diagnosed with multidetector CT (MDCT) [13]. Visualizing extraluminal air on radiograph of the chest and/or abdomen is often the initial clue in diagnosing colonic perforation in patients with acute abdomen [14]. Retroperitoneal air accumulates in a linear fashion along the margins of the kidneys, psoas muscles, and medial subdiaphragmatic region. The differential diagnosis of pneumoretroperitoneum on plain radiography includes pseudopneumoretroperitoneum, pneumoperitoneum, and subcutaneous emphysema, which can be definitively diagnosed with CT. Saeki et al. analyzed the common findings of colonic perforation including free air, ascites, bowel wall thickening, bowel dilation, dirty fat sign, and dirty mass sign. Abdominal X-ray with “dirty mass sign” is a highly specific finding of colonic perforation and appears as localized fecal matter containing a conglomerate of air bubbles in the extraluminal space [1]. MDCT establishes a definitive diagnosis, localizes the perforation, assesses its severity, and provides insight into proper management [[14], [15], [16]]. Treatment of colonic perforation includes surgery with exploratory laparotomy and intra-abdominal lavage along with broad-spectrum antibiotics until a culture is obtained [17]. Other approaches include conservative management and/or drainage of the fluid collection depending on the extent of the perforation.

## Conclusion

Pneumoretroperitoneum is a rare complication of colonic perforation. As seen in this case report, identifying the dirty mass sign on abdominal radiograph can help to rapidly diagnose the etiology of acute abdomen and initiate treatment to prevent complications such as peritonitis and sepsis. MDCT is the best imaging technique to assess the severity of the perforation and inform management of the condition.

## Patient consent

Written informed consent was obtained from the patient for the publication of this case report. This project did not involve any research and no ethical clearance was required.
